# Impact of mitral valve transcatheter edge‐to‐edge repair on haemodynamic parameters in cardiogenic shock

**DOI:** 10.1002/ehf2.15306

**Published:** 2025-04-14

**Authors:** Michal Droppa, Dominik Rath, Philippa Jaeger, Ioannis Toskas, Monika Zdanyte, Andreas Goldschmied, Jürgen Schreieck, Meinrad Gawaz, Tobias Geisler

**Affiliations:** ^1^ Department of Cardiology and Angiology University Hospital of Tübingen Tübingen Germany

**Keywords:** Cardiogenic shock, Heart failure, Mitral valve transcatheter edge‐to‐edge repair

## Abstract

**Background:**

Transcatheter edge‐to‐edge repair (TEER) has been shown to be an effective treatment option for patients experiencing cardiogenic shock (CS) with concomitant high‐grade mitral valve regurgitation. However, haemodynamic changes following M‐TEER have not been thoroughly investigated. Afterload mismatch, leading to the deterioration of haemodynamics subsequent to mitral regurgitation correction, could potentially occur and adversely impact prognosis. Our objective was to analyse the effect of TEER on haemodynamic and echocardiographic parameters in patients with CS.

**Methods and results:**

We conducted a retrospective study of patients undergoing TEER for mitral valve regurgitation in the setting of CS. Haemodynamic and echocardiographic parameters before and after TEER were systematically analysed. A total of 25 patients underwent TEER in the context of CS. All patients were successfully treated with at least of one grade reduction in mitral regurgitation. The median left atrial mean pressure decreased from 23 mmHg (IQR 17–30) to 16 mmHg (IQR 11–20, *P* < 0.01), and the V‐wave decreased from 36 mmHg (IQR 27–44) to 21 mmHg (IQR 14–25, *P* < 0.01) following the procedure. The stroke volume index and cardiac index increased from 25 mL/m^2^ (IQR 18–29) to 34 mL/m^2^ (IQR 25–44, *P* < 0.01) and from 1.90 L/min/m^2^ (IQR 1.41–2.30) to 2.50 L/min/m^2^ (IQR 1.99–2.86, *P* < 0.01), respectively. We did not observe any worsening of the ejection fraction after the procedure. Ten patients (40%) died during their hospital stay.

**Conclusions:**

Our study demonstrates that TEER leads to favourable haemodynamic changes in patients with CS. We observed a significant reduction in left atrial pressure, V‐wave, and an elevation in cardiac index. Importantly, we did not observe any deterioration in left ventricular function following the procedure. This supports the concept of haemodynamic stabilization with TEER in patients with CS and high‐grade mitral regurgitation.

## 1 Background

Cardiogenic shock (CS) is life‐threating condition associated with a high mortality rate of about 50%, despite recent progress in treating heart failure. In CS, low cardiac output due to reduced ejection fraction leads to multiorgan failure. Especially in patients with acute deterioration of chronic heart failure, cardiac remodelling with dilation of the left ventricle can lead to high‐grade mitral regurgitation, which can further reduce stroke volume and thus worsen heart failure. Mitral valve repair could improve haemodynamics and cardiac output; however, it is usually unavailable for critical ill patients due to very high operative risk. Transcatheter edge‐to‐edge repair (TEER) has been shown to be an effective treatment option for patients experiencing cardiogenic shock with concomitant high‐grade mitral valve regurgitation in several observational studies.^1^, ^2^, ^3^, ^4^, ^5^, ^6^, ^7^, ^8^, ^9^, ^10^, ^11^, ^12^, ^13^ However, haemodynamic changes following mitral‐clip implantation have not been thoroughly investigated. Afterload mismatch, leading to the deterioration of haemodynamics subsequent to mitral regurgitation correction, could potentially occur and adversely impact prognosis.^14^

## 2 Aims

Our objective was to analyse haemodynamic and echocardiographic parameters in patients with CS undergoing TEER.

## 3 Methods

We conducted a retrospective single centre study of patients undergoing TEER for mitral valve repair in the setting of CS at our hospital. We analysed all mitral TEER procedures at our centre from January 2012 to November 2023. From the patients' records, we identified those with CS and available haemodynamic data for the analysis. CS was defined according to SCAI SHOCK classification at least in stage C.^15^ Severe MR was defined by echocardiographic criteria according to current guidelines after evaluation by transesophageal echocardiography (TOE).^16^ Data for the analysis were obtained from electronic patients records, and echocardiographic parameters were accessed from the Picture Archiving and Communication System (PACS) of our clinic.

The decision to treat a patient with TEER was made by an interdisciplinary heart team consisting of a cardiologist and cardiac surgeon. TEER was performed in hybrid operating room under general anaesthesia with TOE guidance. Continuous patients monitoring with invasive arterial blood pressure measurement, ECG and oxygen saturation was performed.

Baseline characteristics, including MR aetiology, relevant patient history, comorbidities, supportive medication, and mechanical circulatory support were obtained. The following prespecified parameter were collected for the analysis: MR grade before and after TEER as assessed by TOE during the procedure, mean pressure gradient after the TEER. Explorative procedure outcomes included MR reduction (by at least one grade), procedural mortality, successful deployment and positioning of the device, freedom of emergency surgery or repeated intervention, and the occurrence of major vascular or cardiac structural complications during the hospital stay. In transthoracic echocardiography, ejection fraction and end‐diastolic left ventricular diameter (LVEDD) were measured before and 24–48 h after the TEER procedure. The following haemodynamic parameters were assessed before and after TEER procedure: mean arterial blood pressure measured by the cannulation of a peripheral artery, mean left atrial pressure and V‐wave measured either by pulmonary catheterization with a Swan‐Ganz catheter or directly in the left atrium using the TEER‐device guiding catheter, systolic pulmonary pressure measured either by pulmonary catheterization or indirectly in echocardiography, and cardiac index and stroke volume assessed before and after TEER by the Fick method or in echocardiography as previously described.^17^ Mortality and ICU stay were assessed from patients records.

Data are presented as median and interquartile range. ANOVA for repeated measures was used to analyse changes in studied parameters. For analyses, a two‐tailed *P*‐value < 0.05 was considered statistically significant. Statistical analysis was performed with SPSS version 28.0 (IBM, USA).

The study was approved by the local ethics committee (753/2023B02) and complies with the Declaration of Helsinki and German law for retrospective data analysis.

## 4 Results

**Figure 1:**
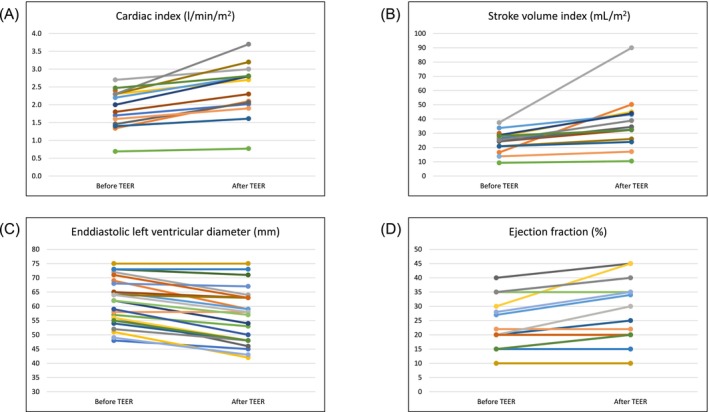
Selected haemodynamic and echocardiographic parameters in patients with cardiogenic shock before and after mitral transcatheter edge‐to‐edge repair (TEER). *P* < 0.01 for all parameters. (A) cardiac index, (B) stroke volume index, (C) end‐diastolic left ventricular diameter, (D) ejection fraction.

A total of 25 patients underwent TEER in the context of CS. Baseline characteristics are listed in *Table* *1*. The median age of the patients was 65 years, and 80% were men. Most of the patient had ischaemic cardiomyopathy, with a median ejection fraction of 20%. The aetiology of mitral regurgitation was secondary due to left ventricular dilatation in 72% of the cases and combined in 28%. One patient experienced acute coronary syndrome complicated by cardiogenic shock and acute mitral regurgitation caused by rapid left ventricular dysfunction following a large anterior myocardial infarction. All other patients presented with acute decompensated heart failure with reduced ejection fraction, leading to cardiogenic shock. All patients were on supportive drugs (vasopressors or inotropes), and 40% were on mechanical cardiac support. All patients were successfully treated with at least a one‐grade reduction in mitral regurgitation, and no patient had high‐grade regurgitation or an elevated gradient through the mitral valve after the procedure (*Table* *2*). There were no device failures or major device‐related complications in any patient during the hospital stay. Haemodynamic and echocardiographic parameters are listed in *Table* *3*. We did not observe a significant change in mean arterial pressure (*P* = 0.06). However, the median left atrial mean pressure and V‐wave decreased from 23 mmHg (IQR 17–30) to 16 mmHg (IQR 11–20) and from 36 mmHg (IQR 27–44) to 21 mmHg (IQR 14–25), respectively (*P* < 0.01 for both parameters). The stroke volume index and cardiac index increased from 25 mL/m^2^ (IQR 18–29) to 34 mL/m^2^ (IQR 25–44) and from 1.90 L/min/m^2^ (IQR 1.41–2.30) to 2.50 L/min/m^2^ (IQR 1.99–2.86), respectively (*P* < 0.01 for both parameters). Pulmonary arterial pressure decreased from 54 mmHg (IQR 43–60) to 47 mmHg (IQR 34–62) after the procedure (*P* = 0.04). In echocardiographic parameters, we observed significant reduction of LVEDD from 62 mm (IQR 55–70) to 58 mm (48–64, *P* < 0.01). We did not observe a deterioration of haemodynamics in any patient after TEER in terms of progressive LV dilatation or worsening of ejection fraction (*Table* *3*, *Figure* *1*).

**Table 1:** Baseline characteristics

Age (years)	65 (57–71)
Sex male/female	20 (80%)/5 (20%)
Diabetes	8 (32%)
Hypertension	18 (72%)
Dyslipidaemia	17 (68%)
Atrial fibrillation	17 (68%)
Coronary artery disease	19 (76%)
Prior MI	18 (72%)
Prior PCI	17 (68%)
Prior CABG	7 (28%)
Ejection fraction (%)	20 (15–29)
Systolic pulmonary pressure (mmHg)	54 (44–60)
Aetiology of mitral regurgitation	
Secondary	18 (72%)
Combined	7 (28%)
Aetiology of cardiogenic shock	
Ischaemic cardiomyopathy	18 (72%)
Acute post‐MI	1 (4%)
Chronic decompensated	17 (68%)
Non‐ischaemic cardiomyopathy	7 (28%)
Supportive drugs	
Vasopressors	25 (100%)
Inotropic substances	21 (84%)
Mechanical cardiac support before/during procedure	10 (40%)
IABP	1 (4%)
Axial flow pump	3 (12%)
ECMO	3 (12%)
ECMO and axial flow pump	3 (12%)

Baseline characteristics of patients with cardiogenic shock undergoing mitral transcatheter edge‐to‐edge repair. Data are presented as medians and interquartile range or absolute number and percentage.

CABG, coronary artery bypass graft; ECMO, extracorporeal membrane oxygenation; IABP, intra‐aortic balloon pump; MI, myocardial infarction; PCI, percutaneous coronary intervention.

**Table 2:** Procedural characteristics

Mitral regurgitation grade	4 (3–4)
Number of clips	2 (1–2)
Abbott MitraClip/Edwards Pascal	22 (88%) /3 (12%)
Procedural success	25 (100%)
Mean pressure gradient after TEER (mmHg)	2 (2–3)

Procedural success – MR reduction (by at least one grade), absence of procedural mortality, successful deployment and positioning of the device, freedom of emergency surgery or repeated intervention, and the occurrence of major vascular or cardiac structural complications during the hospital stay.

TEER, transcatheter edge‐to‐edge repair.

**Table 3:** Haemodynamics and echocardiographic parameters

	Before procedure	After procedure	*P* value
Mitral regurgitation grade (*N* = 25)	4 (3–4)	1 (1–2)	<0.01
Ejection fraction, % (*N* = 25)	20 (15–29)	20 (20–35)	<0.01
LVEDD, mm (*N* = 25)	62 (55–70)	58 (48–64)	<0.01
Haemodynamics			
Mean arterial pressure, mmHg (*N* = 25)	68 (62–74)	71 (66–81)	0.06
Mean left atrial pressure, mmHg (*N* = 25)	23 (17–30)	16 (11–20)	<0.01
V‐wave left atrial, mmHg (*N* = 25)	36 (27–44)	21 (14–25)	<0.01
Systolic pulmonary pressure, mmHg (*N* = 16)	54 (43–60)	47 (34–62)	0.04
Cardiac output, L/min (*N* = 16)	3.67 (2.51–4.35)	4.90 (3.75–5.43)	<0.01
Cardiac index, L/min/m^2^ (*N* = 16)	1.90 (1.41–2.30)	2.50 (1.99–2.86)	<0.01
Stroke volume index, mL/m^2^ (*N* = 16)	25 (18–29)	34 (25–44)	<0.01

Haemodynamics and echocardiographic parameters in patients before and after mitral transcatheter edge‐to‐edge repair.

LVEDD, end‐diastolic left ventricular diameter.

Of the 25 patients in CS, 15 patients recovered and were able to be discharged. Ten patients (40%) died due to persistent cardiogenic shock despite the therapy. Notably, no patient died within 48 hours after the procedure. There were no significant differences in baseline parameters—age, diabetes, aetiology of cardiogenic shock, baseline ejection fraction or LVEDD—between survivors and deceased patients. However, the need for a mechanical cardiac support was significantly associated with mortality (70% of patients requiring mechanical cardiac support died, *P* = 0.03). Patients who survived could be weaned off vasopressors and inotropes very quickly, with a median of 1 day (IQR 1–3). Furthermore, guideline‐directed medical therapy could be initiated after a median of 2 days (IQR 2–4). Most patients were treated with a combination of at least three drugs at discharge: 80% were on ACE inhibitors or AT1 receptor blockers, 73.3% on beta‐blockers, 86.7% on aldosterone antagonists, 13.3% on neprilysin inhibitors and 26.7% on SGLT2 inhibitors.

## 5 Conclusions

In recent years, M‐TEER has been established as the standard therapy for high‐grade MR in patients at high surgical risk.^18^, ^19^, ^20^ In patients with secondary MR and heart failure, there are conflicting results from randomized studies. While the COAPT and RESHAPE‐HF2 studies showed a significant reduction in hospitalization for heart failure and reduced mortality with TEER,^21^, ^22^ the MITRA‐FR study did not show a benefit.^23^ Recently, the MATTERHORN trial demonstrated the clinical non‐inferiority of M‐TEER compared to surgical mitral valve repair in heart failure, with fewer major adverse events.^24^

Patients with cardiogenic shock represent a challenging population with high mortality and limited therapeutic options. The ongoing randomized trial CAPITAL MINOS^25^ will investigate the role of M‐TEER in inotrope‐dependent CS. However, data from randomized studies are currently lacking. Evidence from multicentric registries and meta‐analyses suggests that M‐TEER may be an effective treatment option for patients with acute post‐myocardial infarction MR, showing favourable outcomes in those with or without CS.^9^, ^10^, ^11^, ^12^ Additionally, several observational studies, including patients with other etiologies of CS, such as decompensated chronic heart failure, indicate that M‐TEER may be a viable option to improve the patient outcomes.^1^, ^2^, ^3^, ^4^, ^5^, ^6^, ^7^, ^8^, ^13^ The MitraBridge registry showed favourable outcomes of M‐TEER in patients with end‐stage heart failure awaiting heart transplantation.^26^

Despite the beneficial effects on cardiac output due to reduction of the regurgitant fraction after TEER, negative effects on left ventricular function are a concern. The deterioration of ventricular function after surgical valve replacement was described many years ago.^27^, ^28^ Due to the increase in afterload following the abrupt reduction of MR, left ventricular dilatation and worsening of the left ventricular ejection fraction can be observed in a small group of patients after surgical repair. Afterload mismatch has also been described in some observational studies after TEER^29^, ^30^; however, patients with CS have not been studied extensively.

In our study, we observed favourable haemodynamic changes after clip placement in patients with CS. All patients were treated effectively, which was accompanied by a significant reduction in mean left atrial pressure and V‐wave, as well as a decrease in pulmonary pressure. Consequently, cardiac output increased significantly in all patients. Importantly, we did not observe any deterioration in left ventricular function following the procedure, in terms of a reduction in ejection fraction or an increase in left ventricular diameter. Despite the therapy, we observed a 40% mortality rate in our study. Previous observational studies reported slightly lower mortality rate: approximately 20% for patients with acute MR following myocardial infarction^12^ and around 30% for those with decompensated heart failure.^5^, ^6^, ^8^ In our population, only one patient had MR aetiology due to acute myocardial infarction. All other patients experienced CS in decompensated heart failure from other causes mostly severe chronic heart failure. Furthermore, the use of mechanical support in our study was high. Therefore, the high mortality observed in our study likely reflects both the underlying aetiology and the severity of the condition in patients with advanced stages of CS. All patients who died succumbed to multiorgan failure in context of the CS. Nevertheless, no patient died within 48 hours after the TEER due to worsening of ventricle function or procedure complication. Patients who survived could be quickly weaned off vasopressors and inotropes, and the majority could be initiated on combination therapy for heart failure. This supports the concept of haemodynamic stabilization with TEER in patients with cardiogenic shock and high‐grade mitral regurgitation. Optimized haemodynamics may allow for cardiac recovery, weaning from vasopressors and inotropes, up‐titration of guideline‐directed medical therapy,^31^ or even bridging to implantation of a left ventricular assist device in selected patients with end‐stage cardiac failure.^26^

The limitations of this study should be acknowledged. It was a retrospective study with a small patient cohort and lacked a control population. Procedures were performed in a wide time frame, during which different generation devices were used, and operator experience likely improved over time. Mortality was high among the study population. Furthermore, the use of mechanical circulatory support and inotropes was not standardized, which may have influenced the haemodynamic outcomes. There was also heterogeneity in the evaluation of haemodynamic parameters, as pulmonary catheterization and echocardiography were both used to measure pulmonary pressure and cardiac index. However, the same method was consistently applied to each individual before and after the procedure.

## Conflict of interest

JS received honoraria/speaker fees and travel grants by Edwards Lifescience. TG received honoraria/speaker fees, travel grants and institutional research grants by Edwards Lifescience. MD, DR, PJ, IT, MZ, AG, and MG declare no conflict of interest.

## References

[1] Falasconi G , Melillo F , Pannone L , Adamo M , Ronco F , Latib A , et al. Use of edge‐to‐edge percutaneous mitral valve repair for severe mitral regurgitation in cardiogenic shock: a multicenter observational experience (MITRA‐SHOCK study). Catheter Cardiovasc Interv Off J Soc Card Angiogr Interv 2021;98:E163–E170. doi:10.1002/ccd.29683 33797142

[2] Flint K , Brieke A , Wiktor D , Carroll J . Percutaneous edge‐to‐edge mitral valve repair may rescue select patients in cardiogenic shock: findings from a single center case series. Catheter Cardiovasc Interv Off J Soc Card Angiogr Interv. 2019;94:E82–E87. doi:10.1002/ccd.28089 30677212

[3] Cheng R , Dawkins S , Hamilton MA , Makar M , Hussaini A , Azarbal B , Patel JK , Kobashigawa JA , Trento A , Makkar RR , Kar S Percutaneous mitral repair for patients in cardiogenic shock requiring inotropes and temporary mechanical circulatory support. JACC Cardiovasc Interv 2019;12:2440–2441. doi:10.1016/j.jcin.2019.05.042 31806229

[4] Garcia S , Alsidawi S , Bae R , Cavalcante J , Eckman P , Gössl M , Steffen R , Sun B , Schmidt CW , Sorajja P Percutaneous mitral valve repair with MitraClip in inoperable patients with severe mitral regurgitation complicated by cardiogenic shock. J Invasive Cardiol 2020;32:228–231. doi:10.25270/jic/19.00453. 32385191

[5] Jung RG , Simard T , Kovach C , Flint K , Don C , Di SP , et al. Transcatheter mitral valve repair in cardiogenic shock and mitral regurgitation. JACC Cardiovasc Interv 2021;14:1–11. doi:10.1016/j.jcin.2020.08.037 33069653

[6] Tang GHL , Estevez‐Loureiro R , Yu Y , Prillinger JB , Zaid S , Psotka MA . Survival sollowing edge‐to‐edge transcatheter mitral valve repair in patients with cardiogenic shock: a nationwide analysis. J Am Heart Assoc 2021;10:e019882. doi:10.1161/JAHA.120.019882 33821669 PMC8174169

[7] Martinez‐Gomez E , McInerney A , Tirado‐Conte G , de Agustin JA , Jimenez‐Quevedo P , Escudero A , et al. Percutaneous mitral valve repair with MitraClip device in hemodynamically unstable patients: a systematic review. Catheter Cardiovasc Interv Off J Soc Card Angiogr Interv. 2021;98:E617–E625. doi:10.1002/ccd.29703 33856097

[8] Simard T , Vemulapalli S , Jung RG , Vekstein A , Stebbins A , Holmes DR , Czarnecki A , Hibbert B , Alkhouli M Transcatheter edge‐to‐edge mitral valve repair in patients with severe mitral regurgitation and cardiogenic shock. J Am Coll Cardiol 2022;80:2072–2084. doi:10.1016/j.jacc.2022.09.006 36126766

[9] Estévez‐Loureiro R , Shuvy M , Taramasso M , Benito‐Gonzalez T , Denti P , Arzamendi D , et al. Use of MitraClip for mitral valve repair in patients with acute mitral regurgitation following acute myocardial infarction: effect of cardiogenic shock on outcomes (IREMMI registry). Catheter Cardiovasc Interv Off J Soc Card Angiogr Interv. 2021;97:1259–1267. doi:10.1002/ccd.29552 33600072

[10] Haberman D , Estévez‐Loureiro R , Benito‐Gonzalez T , Denti P , Arzamendi D , Adamo M , et al. Safety and seasibility of MitraClip implantation in patients with acute mitral regurgitation after recent myocardial infarction and severe left ventricle dysfunction. J Clin Med 2021;10:1819. doi:10.3390/jcm10091819 33921996 PMC8122348

[11] Estévez‐Loureiro R , Lorusso R , Taramasso M , Torregrossa G , Kini A , Moreno PR . Management of severe mitral regurgitation in patients with acute myocardial infarction: JACC focus seminar 2/5. J Am Coll Cardiol 2024;83:1799–1817. doi:10.1016/j.jacc.2023.09.840 38692830

[12] Calì F , Pagnesi M , Pezzola E , Montisci A , Metra M , Adamo M . Transcatheter edge‐to‐edge mitral valve repair for post‐myocardial infarction papillary muscle rupture and acute heart failure: a systematic review. Catheter Cardiovasc Interv Off J Soc Card Angiogr Interv. 2023;102:138–144. doi:10.1002/ccd.30682 37161909

[13] Shuvy M , Maisano F . Evolving indications for transcatheter mitral edge‐to‐edge repair. EuroIntervention J Eur Collab Work Group Interv Cardiol Eur Soc Cardiol 2024;20:e230–e238. doi:10.4244/EIJ-D-23-00700 PMC10870010 38389473

[14] Tang GHL , Cohen M , Dutta T , Undemir C . Afterload mismatch after transcatheter mitral valve repair with MitraClip for degenerative mitral regurgitation in acute cardiogenic shock. Catheter Cardiovasc Interv Off J Soc Card Angiogr Interv. 2018;92:E168–E171. doi:10.1002/ccd.27019 28303686

[15] Naidu SS , Baran DA , Jentzer JC , Hollenberg SM , van Diepen S , Basir MB , et al. SCAI SHOCK stage classification expert consensus update: a review and incorporation of validation studies: this statement was endorsed by the American College of Cardiology (ACC), American College of Emergency Physicians (ACEP), American Heart Association (AHA), European Society of Cardiology (ESC) Association for Acute Cardiovascular Care (ACVC), International Society for Heart and Lung Transplantation (ISHLT), Society of Critical Care Medicine (SCCM), and Society of Thoracic Surgeons (STS) in December 2021. J Am Coll Cardiol 2022;79:933–946. doi:10.1016/j.jacc.2022.01.018 35115207

[16] Vahanian A , Beyersdorf F , Praz F , Milojevic M , Baldus S , Bauersachs J , et al. 2021 ESC/EACTS guidelines for the management of valvular heart disease: developed by the task force for the management of valvular heart disease of the European Society of Cardiology (ESC) and the European Association for Cardio‐Thoracic Surgery (EACTS). Eur Heart J 2022;43:561–632. doi:10.1093/eurheartj/ehab395 34453165

[17] Patzelt J , Zhang Y , Magunia H , Jorbenadze R , Droppa M , Ulrich M , et al. Immediate increase of cardiac output after percutaneous mitral valve repair (PMVR) determined by echocardiographic and invasive parameters: Patzelt: increase of cardiac output after PMVR. Int J Cardiol 2017;236:356–362. doi:10.1016/j.ijcard.2016.12.190 28185701

[18] Feldman T , Kar S , Elmariah S , Smart SC , Trento A , Siegel RJ , et al. Randomized comparison of percutaneous repair and surgery for mitral regurgitation: 5‐year results of EVEREST II. J Am Coll Cardiol 2015;66:2844–2854. doi:10.1016/j.jacc.2015.10.018 26718672

[19] Buzzatti N , Van Hemelrijck M , Denti P , Ruggeri S , Schiavi D , Scarfò IS , et al. Transcatheter or surgical repair for degenerative mitral regurgitation in elderly patients: a propensity‐weighted analysis. J Thorac Cardiovasc Surg 2019;158:86–94.e1. doi:10.1016/j.jtcvs.2019.01.023 30797588

[20] Sorajja P , Vemulapalli S , Feldman T , Mack M , Holmes DR , Stebbins A , et al. Outcomes with transcatheter mitral valve repair in the United States: an STS/ACC TVT registry report. J Am Coll Cardiol 2017;70:2315–2327. doi:10.1016/j.jacc.2017.09.015 29096801

[21] Stone Gregg W. , Lindenfeld JoAnn , Abraham William T. , Kar Saibal , Lim D. Scott , Mishell Jacob M. , et al. Transcatheter mitral‐valve repair in patients with heart failure. N Engl J Med 2018;379:2307–2318. doi:10.1056/NEJMoa1806640 30280640

[22] Anker SD , Friede T , von Bardeleben RS , Butler J , Khan MS , Diek M , et al. Transcatheter valve repair in heart failure with moderate to severe mitral regurgitation. N Engl J Med 2024;391:1799–1809. doi:10.1056/NEJMoa2314328 39216092

[23] Obadia JF , Messika‐Zeitoun D , Leurent G , Iung B , Bonnet G , Piriou N , et al. Percutaneous repair or medical treatment for secondary mitral regurgitation. N Engl J Med 2018;379:2297–2306. doi:10.1056/NEJMoa1805374 30145927

[24] Baldus S , Doenst T , Pfister R , Gummert J , Kessler M , Boekstegers P , et al. Transcatheter repair versus mitral‐valve surgery for secondary mitral regurgitation. N Engl J Med 2024;391:1787–1798. doi:10.1056/NEJMoa2408739 39216093

[25] Parlow S , Di Santo P , Jung RG , Fam N , Czarnecki A , Horlick E , et al. Transcatheter mitral valve repair for inotrope dependent cardiogenic shock ‐ design and rationale of the CAPITAL MINOS trial. Am Heart J 2022;254:81–87. doi:10.1016/j.ahj.2022.08.008 36002047

[26] Munafò AR , Scotti A , Estévez‐Loureiro R , Adamo M , Hernàndez AP , Peregrina EF , et al. 2‐year outcomes of MitraClip as a bridge to heart transplantation: the international MitraBridge registry. Int J Cardiol 2023;390:131139. doi:10.1016/j.ijcard.2023.131139 37355239

[27] Schuler G , Peterson KL , Johnson A , Francis G , Dennish G , Utley J , et al. Temporal response of left ventricular performance to mitral valve surgery. Circulation 1979;59:1218–1231. doi:10.1161/01.CIR.59.6.1218 436214

[28] Ross J. Afterload mismatch in aortic and mitral valve disease: implications for surgical therapy. J Am Coll Cardiol 1985;5:811–826. doi:10.1016/S0735-1097(85)80418-6 3882814

[29] Jogani S , Van de Heyning CM , Paelinck BP , De Bock D , Mertens P , Heidbuchel H , et al. Afterload mismatch after MitraClip implantation: intraoperative assessment and prognostic implications. J Invasive Cardiol 2020;32:88–93, doi:10.25270/jic/19.00336. 32024805

[30] Melisurgo G , Ajello S , Pappalardo F , Guidotti A , Agricola E , Kawaguchi M , et al. Afterload mismatch after MitraClip insertion for functional mitral regurgitation. Am J Cardiol 2014;113:1844–1850. doi:10.1016/j.amjcard.2014.03.015 24837263

[31] Adamo M , Tomasoni D , Stolz L , Stocker TJ , Pancaldi E , Koell B , et al. Impact of transcatheter edge‐to‐edge mitral valve repair on guideline‐directed medical therapy uptitration. JACC Cardiovasc Interv 2023;16:896–905. doi:10.1016/j.jcin.2023.01.362 37100553

